# Supplementary material for: CABS-dock web server for the flexible docking of peptides to proteins without prior knowledge of the binding site
Source: Nucleic Acids Res. 2015 May 5;43(Web Server issue):W419–24. doi: 10.1093/nar/gkv456 (PMC4489223; doi:10.1093/nar/gkv456)
Supplement: SUPPLEMENTARY DATA [file supp_43_W1_W419__index.html]

CABS-dock web server for the flexible docking of peptides to proteins without prior knowledge of the binding site — CABS-dock web server for the flexible docking of peptides to proteins without prior knowledge of the binding site — SUPPLEMENTARY DATA 

# CABS-dock web server for the flexible docking of peptides to proteins without prior knowledge of the binding site

## SUPPLEMENTARY DATA

**Files in this Data Supplement:**

- SUPPLEMENTARY DATA
