# Supplementary material for: CABS-dock web server for the flexible docking of peptides to proteins without prior knowledge of the binding site
Source: Nucleic Acids Res. 2015 May 5;43(Web Server issue):W419–24. doi: 10.1093/nar/gkv456 (PMC4489223; doi:10.1093/nar/gkv456)
Supplement: SUPPLEMENTARY DATA [file supp_gkv456_nar-00687-web-b-2015-File005.pdf]

Supplementary data

Summary of the CABS force-field

Detailed description of the CABS force-field can be found in the ref. [Kolinski, A. (2004) Protein modeling and structure prediction with a reduced representation. *Acta biochimica Polonica*, **51**, 349-371]. For readers' convenience it is summarized below.

The CABS force field is a knowledge-based statistical potential. It has been derived from regularities observed in known protein structures (deposited in the Protein Data Bank). Total energy of the modeled system consists of the following terms:

- **Short-range (along the sequence) sequence-independent interactions**
  This term is in fact a set of conditions and constraints imposed on the virtual Cα-Cα bonds to enforce protein-like behavior of the otherwise very flexible Cα trace (e.g. 3.28 Å< $||\mathbf{R}_i-\mathbf{R}_{i+1}||$ < 4.27 Å, where $\mathbf{R}_i$ is a coordinate vector of the Cα atom in the i-th residue).
- **Short-range (along the sequence) sequence-dependent interactions**
  This potential is responsible for local conformation of short (3-5 amino acids) fragments of the protein chain. It depends on types of the amino acids which compose the fragment, local secondary structure and chirality. It is stored in histograms of distances between i-th and i+2nd, i+3rd and i+4th Cα atoms. For a given fragment the energetic reward is exponentially proportional to the frequency of occurrence in known structures of the respective Cα-Cα distance observed in that fragment.
- **Long-range (along the sequence) sequence-independent interactions**
  This potential includes the terms responsible for the short-distance repulsion (excluded volume) and a set of geometric terms which define hydrogen bonds between backbone atoms
- **Long-range (along the sequence) sequence-dependent interactions**
  This is a two-body, context-dependent contact potential responsible for side chains' interactions. It is stored in multi-dimensional arrays which contain cut-off values of distances between contacting side chain pseudo atoms and energetic reward/penalty for contact occurrence. The arrays are indexed by the types of interacting residues (ALA, GLY, …), local conformation of the backbone in both interacting residues (compact helix- and turn-like or open sheet- and coil-like) and relative orientation of the side chains of the interacting residues (parallel, intermediate and anti-parallel). This potential implicitly considers also the electrostatic interactions, disulphide bridges, hydrophobic effect of the solvent and hydrogen bonds between side chain atoms.
- **Centrosymmetric potential**
  This term is responsible for the sphere-like shape of the protein. Diameter of the protein is estimated from the number of residues it contains. Energetic reward/penalty per residue depends on its distance from the proteins' geometric center i.e. polar resides are supposed to be located on the surface of the protein and non-polar constitute its hydrophobic core.

Full set of the CABS parameters is available from the laboratory website http://biocomp.chem.uw.edu.pl/

Figure S1. Flow-chart of the CABS-dock protocol.

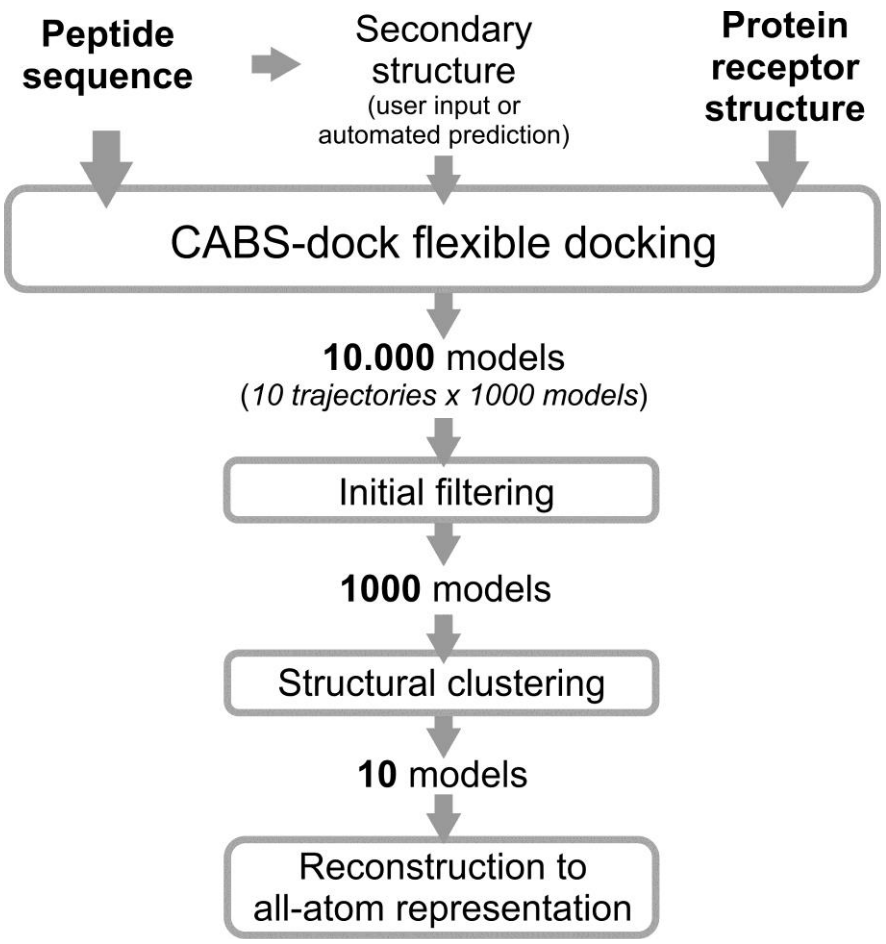

Table S1. CABS-dock performance for 103 bound cases (listed in rows) in 3 independent prediction runs (shown in separate columns). The table shows the lowest ligand-RMSD values (calculated on the peptide only after superimposition of the receptor structures) among: all 10,000 models (all), top 1000 models (top 1k, selected during filtering and clustering procedure), top 100 models (top 100, selected through further clustering) and top 10 final models (top 10). Last column shows the lowest RMSD values obtained in three prediction runs.

receptor	receptor	peptide	Run 1				Run 2				Run 3				best from all runs			
pdb id	length (AA)	length (AA)	all	top 1k	top 100	top 10	all	top 1k	top 100	top 10	all	top 1k	top 100	top 10	all	top 1k	top 100	top 10
1awr	164	6	1.51	2.48	2.92	2.83	1.99	1.99	2.82	5.52	1.86	1.89	2.83	3.97	1.51	1.89	2.82	2.83
1ce1	431	8	1.73	2.14	3.34	4.23	2.76	3.31	3.76	5.45	3.12	3.48	4.32	3.86	1.73	2.14	3.34	3.86
1cka	57	9	3.45	3.54	4.73	5.03	3.4	3.73	4.36	5.08	3.09	3.37	4.03	4.45	3.09	3.37	4.03	4.45
1czy	168	7	2.68	4.51	11.58	18.99	1.91	2.21	2.4	3.05	2.39	3.37	6.06	11.05	1.91	2.21	2.4	3.05
1d4t	104	11	4.8	5.61	7.37	8.17	1.97	1.97	2.2	2.63	2.15	2.97	3.18	3.85	1.97	1.97	2.2	2.63
1ddv	104	6	2.52	2.94	3.97	3.82	3.24	3.88	4.75	4.07	3.08	3.53	4.7	6.92	2.52	2.94	3.97	3.82
1dkx	219	7	8.49	8.49	9.11	8.84	7.93	7.93	8.19	9.4	9.59	9.74	10.29	12.78	7.93	7.93	8.19	8.84
1eg4	260	13	6.73	8.61	8.97	11.08	9.57	11.38	12.26	16.18	6.57	13.12	13.39	13.7	6.57	8.61	8.97	11.08
1elw	117	8	1.96	1.96	2.53	3.8	2.18	2.94	3.3	4.87	2.56	2.56	3.38	4.57	1.96	1.96	2.53	3.8
1er8	330	8	6.33	6.92	6.92	6.92	7.11	7.11	7.78	8.68	6.39	6.39	6.9	8.9	6.33	6.39	6.9	6.92
1gyb	122	5	4.53	8.62	11.55	16.66	4.79	7.5	14.76	15.15	4.98	7.67	10.99	14.83	4.53	7.5	10.99	14.83
1h6w	151	10	6.1	18.63	19.07	25.35	8.22	8.22	19.27	20.32	9.46	10.2	12.11	25.23	6.1	8.22	12.11	20.32
1hc9	74	13	6.87	10.59	12.91	14.26	6.21	6.52	7.17	7.94	6.67	11.81	13.69	14.5	6.21	6.52	7.17	7.94
1i8k	225	10	3.6	4	4.04	8.5	5.53	5.82	7.2	7.64	4.92	5.5	6.94	7.8	3.6	4	4.04	7.64
1iak	367	13	3.4	4.19	4.19	5.13	5.56	8.19	10.16	18.04	3.98	3.98	5.2	5.85	3.4	3.98	4.19	5.13
1ihj	94	5	2.58	3.71	4.42	6.99	2.7	2.72	5.29	6.5	2.17	4.84	5.67	7.03	2.17	2.72	4.42	6.5
1jbu	239	15	7.73	9.67	13.45	19.68	9.39	14.52	14.52	26.58	7.39	13.46	21.87	24.87	7.39	9.67	13.45	19.68
1jd5	105	8	2.85	2.85	5.88	16.12	5.31	13.06	15.47	20.07	6.04	9	11.69	15.18	2.85	2.85	5.88	15.18
1jwg	140	5	2.53	3.48	4.41	5.27	2.29	2.29	3.92	6.75	2.38	2.38	4.21	5.87	2.29	2.29	3.92	5.27
1kl3	120	6	2.6	3.51	5.14	5.33	2.91	4.33	4.82	5.92	2.45	4.29	4.92	5.21	2.45	3.51	4.82	5.21
1klu	369	15	3.57	3.89	4.79	5.86	3.64	3.91	4.5	5.3	3.75	3.8	5.02	6.27	3.57	3.8	4.5	5.3
1lvm	229	7	6.13	7.88	17.09	13.47	5.68	9.59	12.52	14.37	5.42	6.07	14.41	15.07	5.42	6.07	12.52	13.47
1mfg	95	9	4.62	4.86	6.78	9.49	2.99	4.6	10.1	7.37	3.71	3.71	7.25	6.78	2.99	3.71	6.78	6.78
1mvu	333	13	4.26	4.26	5.47	9.85	3.74	4.33	4.89	5.29	3.29	4.84	6.58	7.77	3.29	4.26	4.89	5.29
1n12	136	11	8.43	8.43	9.4	12.11	8.31	8.31	8.72	10.46	9.46	9.89	12.78	14.93	8.31	8.31	8.72	10.46
1n7f	86	8	4.18	7.13	7.81	9.15	4.22	7.07	12.76	12.42	4.72	6.2	10.91	13.1	4.18	6.2	7.81	9.15
1nln	203	11	3.34	3.62	3.97	5.23	5.57	7.07	8.61	12.81	3.07	3.7	3.74	12.51	3.07	3.62	3.74	5.23
1nq7	244	10	2.85	2.85	2.85	8.31	1.03	1.03	1.11	4.66	1.17	1.17	2.47	2.73	1.03	1.03	1.11	2.73
1ntv	152	10	3.14	3.14	5.92	10	2.89	2.89	3.71	5.28	3.6	4.3	5.39	14.3	2.89	2.89	3.71	5.28
1nvr	264	5	5.24	6.2	9.11	8.68	2.83	2.83	6.39	7.5	1.68	1.68	4.06	4.15	1.68	1.68	4.06	4.15
1nx1	173	11	3.03	3.2	3.7	4.2	2.82	2.98	3.26	4.68	2.92	2.94	3.33	4.95	2.82	2.94	3.26	4.2

receptor pdb id	receptor length (AA)	peptide length (AA)	Run 1				Run 2				Run 3				best from all runs			
			all	top 1k	top 100	top 10	all	top 1k	top 100	top 10	all	top 1k	top 100	top 10	all	top 1k	top 100	top 10
1oai	59	9	3.81	4.86	5.27	6.84	3.04	3.56	5.54	6.68	3.23	4.75	6.18	6.53	3.04	3.56	5.27	6.53
1ou8	106	8	4.17	4.17	5.93	9.04	4.86	7.38	9.21	10.79	2.98	3.97	5.65	5.56	2.98	3.97	5.65	5.56
1pz5	435	8	4.58	4.58	5.8	5.63	4.69	5.18	5.32	5.22	5.02	5.97	7.73	8.22	4.58	4.58	5.32	5.22
1qkz	219	10	5	6.45	10.31	15.24	6.71	7.72	11.97	13.27	6.96	6.96	6.96	10.69	5	6.45	6.96	10.69
1rxz	245	11	6.76	6.76	11.68	13.38	4.46	5.22	8.5	11.28	3.54	3.9	6.16	6.11	3.54	3.9	6.16	6.11
1se0	97	7	6.16	7.78	11.06	17.95	4.05	4.05	9.08	7.55	5.34	7.02	9	9.06	4.05	4.05	9	7.55
1sfi	223	14	7.8	7.8	10.63	13.04	7.18	7.18	7.36	7.77	6.74	8.53	8.78	11.51	6.74	7.18	7.36	7.77
1ssh	60	11	3	4.79	5.35	7.05	4.19	4.67	5.44	5.41	3.74	4.32	5.33	5.68	3	4.32	5.33	5.41
1svz	232	6	2.98	2.98	3.82	6.82	2.17	2.17	3.77	5.13	2.8	4.43	5.05	5.19	2.17	2.17	3.77	5.13
1t4f	88	9	2.59	3.1	3.82	4.58	2.81	2.88	2.93	3.02	2.78	2.79	3.43	4.23	2.59	2.79	2.93	3.02
1t7r	250	10	1.94	2.15	2.52	3.35	1.61	2.05	2.05	2.13	1.73	1.92	2.92	1.88	1.61	1.92	2.05	1.88
1tp5	115	6	4.28	7.19	9.28	8.47	1.68	1.73	2.64	3.57	1.15	1.45	2.46	3.76	1.15	1.45	2.46	3.57
1tw6	95	6	3.31	3.93	10.22	8.47	3.27	5.75	10.99	17.83	4.33	7.61	11.44	7.22	3.27	3.93	10.22	7.22
1u00	227	9	8.89	8.89	10.59	11.43	9.67	10.85	11.07	11.86	8.95	9.28	9.28	11.21	8.89	8.89	9.28	11.21
1u8i	441	7	5.85	13.62	19.63	19.99	6.5	9.66	13.42	13.84	5.75	9.79	10.1	16.59	5.75	9.66	10.1	13.84
1u9l	138	7	10.81	10.81	11.61	12.46	8.77	8.77	8.96	9.25	10.06	10.06	10.76	10.64	8.77	8.77	8.96	9.25
1uj0	58	9	3.44	3.53	4.29	5.19	3.49	3.49	4.57	4.83	4.1	4.71	4.9	5.37	3.44	3.49	4.29	4.83
1vzq	250	6	2.98	4.02	6.61	10.51	2.76	4.5	5.03	6.71	3.73	5.85	6.35	7.21	2.76	4.02	5.03	6.71
1w9e	164	5	3.77	6.17	11.09	17.41	5.4	5.76	9.04	16.41	4.19	13.05	16.34	17.86	3.77	5.76	9.04	16.41
1x2r	290	9	4.95	5.02	5.04	5.1	2.89	4.85	6.54	7.13	3.89	5	5.78	7.1	2.89	4.85	5.04	5.1
1xoc	504	9	19.49	19.49	20.94	21.66	17.64	17.97	18.06	20.33	18.15	18.15	18.15	19.29	17.64	17.97	18.06	19.29
1ymt	235	10	2.98	2.99	4.12	5.98	2.54	2.77	2.89	3.28	2.79	3.53	3.72	4.54	2.54	2.77	2.89	3.28
1yph	228	10	4.23	5.19	5.19	7.27	4.03	4.03	4.66	4.63	3.27	12.4	13.23	13.44	3.27	4.03	4.66	4.63
1yuc	240	14	3.29	5.35	6.96	7.92	2.89	3.68	5.25	6.13	4.83	8.22	12.26	13	2.89	3.68	5.25	6.13
1ywo	55	10	2.97	5.09	5.98	7.62	3.95	4.25	6.8	8.07	4.21	4.6	6.01	6.46	2.97	4.25	5.98	6.46
1z9o	238	9	4.61	6.68	7.19	8.37	5.18	6.44	6.83	8.28	5.12	6.03	7.31	8.63	4.61	6.03	6.83	8.28
1zuk	130	11	2.93	3.48	4.53	5.19	3.18	3.18	4.58	4.43	3.2	3.38	3.64	4.46	2.93	3.18	3.64	4.43
2a3i	253	12	3.59	4.14	5.31	5.25	1.7	2.56	3.54	4.02	1.68	2.1	2.1	5.49	1.68	2.1	2.1	4.02
2ai4	111	8	5.94	8.48	8.51	9.49	9.6	11.5	14.81	15.92	7.93	11	13.87	14.01	5.94	8.48	8.51	9.49
2ak5	64	8	3.59	3.6	4.79	6.69	4.29	4.29	6.56	6.57	3.57	3.57	5.23	5.38	3.57	3.57	4.79	5.38
2b1z	238	9	0.91	1.15	1.31	1.5	2.28	2.51	2.52	3.46	0.82	1.05	1.18	1.16	0.82	1.05	1.18	1.16
2b9h	337	12	5.19	9.46	9.7	10.71	2.57	2.9	3.11	2.96	4.53	4.53	14	21.87	2.57	2.9	3.11	2.96
2bba	185	14	4.08	4.79	6.87	7.1	3.53	3.65	5.17	5.26	3.91	4.58	5.41	5.57	3.53	3.65	5.17	5.26
2c3i	266	8	6.09	7.29	8.93	11.16	5.12	6.69	8.61	11.37	3.16	6.05	9.94	11.43	3.16	6.05	8.61	11.16
2cch	256	12	6.04	7.77	10.11	12.51	4.38	5.26	11.49	11.47	3.36	7.15	11.11	26.01	3.36	5.26	10.11	11.47

receptor pdb id	receptor length (AA)	peptide length (AA)	Run 1				Run 2				Run 3				best from all runs			
			all	top 1k	top 100	top 10	all	top 1k	top 100	top 10	all	top 1k	top 100	top 10	all	top 1k	top 100	top 10
2d0n	56	9	3.41	3.66	4.42	6.02	3.27	3.67	4.23	4.68	2.64	2.64	4.22	5.64	2.64	2.64	4.22	4.68
2d5w	602	5	18.64	19.17	19.48	21.23	17.99	18.59	20.08	20.39	18.52	18.52	19.65	20.13	17.99	18.52	19.48	20.13
2ds8	41	6	4.97	4.97	10.05	9.46	3.25	3.25	8.87	9.77	2.15	2.15	5.45	9.42	2.15	2.15	5.45	9.42
2dze	320	6	2.39	2.71	2.71	3.13	2.82	2.97	3.23	3.59	7.08	7.45	7.8	8.58	2.39	2.71	2.71	3.13
2fgr	332	8	6.29	13.42	14.19	15.87	9.8	13.72	14.8	16.07	4.96	13.75	13.75	16.49	4.96	13.42	13.75	15.87
2fmf	128	13	2.87	2.87	7.07	7.5	4.46	4.64	6.09	7.18	4.77	5.14	5.92	6.43	2.87	2.87	5.92	6.43
2fnt	198	7	0.76	0.8	0.8	1.12	4.94	4.94	4.98	5.68	8.72	8.72	8.72	10.73	0.76	0.8	0.8	1.12
2foj	137	7	3.29	3.73	3.94	5.97	2.65	2.92	4.34	4.4	5.38	5.38	8.06	13.62	2.65	2.92	3.94	4.4
2fvj	258	10	1.62	1.82	1.82	2.43	1.46	1.46	1.46	3.49	1.73	2.86	3.31	3.66	1.46	1.46	1.46	2.43
2h9m	304	5	1.79	1.8	1.8	4.42	3.1	3.1	3.43	4.96	1.36	3	3.22	4.86	1.36	1.8	1.8	4.42
2ho2	33	10	4.32	6.62	8.82	15.21	3.94	4.89	6.48	7.04	3.8	5.63	6.33	5.59	3.8	4.89	6.33	5.59
2hpl	100	5	1.28	2.66	3.45	3.94	1.99	2.34	4.06	5.22	1.76	2.08	4	5.16	1.28	2.08	3.45	3.94
2ipu	442	7	5.04	5.5	6.47	8.75	7.4	7.57	9.74	17.13	5.42	7.49	11.56	10.82	5.04	5.5	6.47	8.75
2iv9	469	9	5.84	7.49	8.44	8.45	3.91	4.1	7.82	8.3	3.06	4.9	6.59	6.73	3.06	4.1	6.59	6.73
2j6f	57	8	2.96	5.25	11.17	11.37	3.33	3.49	3.95	5.29	2.98	3.73	4.42	5.37	2.96	3.49	3.95	5.29
2jam	279	6	4.98	7.86	12.7	15.99	4.34	7.51	11.81	17.2	6.16	8.41	12.8	13.62	4.34	7.51	11.81	13.62
2o02	224	14	3.99	5.54	6.41	6.68	3.97	4.08	4.86	4.92	3.54	3.71	4	5.19	3.54	3.71	4	4.92
2o4j	240	12	1.49	2.26	2.41	2.81	1.54	2.02	2.69	2.95	6.07	7.81	11.78	12.76	1.49	2.02	2.41	2.81
2o9v	67	10	3.64	3.85	5.4	6.6	3.76	4.35	5.36	9.44	3.38	3.73	4.6	5.39	3.38	3.73	4.6	5.39
2otu	233	11	4.33	4.33	16.41	15.01	3.15	4.24	6.09	8.79	4.57	7.24	16.38	21.54	3.15	4.24	6.09	8.79
2p0w	319	15	5.88	7.98	7.98	14.35	6.05	14.01	14.57	15.25	6.26	6.26	6.38	15.07	5.88	6.26	6.38	14.35
2p1k	87	11	3.52	4.76	5.27	5.38	6.33	9.82	10	10.33	2.47	2.47	3.06	4.08	2.47	2.47	3.06	4.08
2p1t	211	10	0.97	1.34	1.35	1.37	1.38	1.61	2.15	2.79	0.9	0.9	1.3	1.44	0.9	0.9	1.3	1.37
2p54	267	12	3.35	3.35	3.54	3.96	3.07	3.07	3.61	3.85	1.66	2.25	2.61	2.02	1.66	2.25	2.61	2.02
2puy	60	10	6.13	7.84	18.61	21.21	6.3	6.3	10.36	10.35	3.63	3.63	8.7	12.44	3.63	3.63	8.7	10.35
2pv2	206	12	2.27	2.27	3.6	3.77	3.26	3.81	4.73	5.82	1.72	1.72	1.72	4.43	1.72	1.72	1.72	3.77
2qos	173	11	3.11	4.16	6.57	6.88	3.53	4.83	5.87	6.79	3.17	3.94	4.65	5.76	3.11	3.94	4.65	5.76
2r7g	337	10	1.47	1.62	1.62	3.33	2.34	2.85	3.53	4.97	2.94	3.7	4.03	5.09	1.47	1.62	1.62	3.33
2v3s	96	6	2.42	2.42	3.48	8.89	1.83	1.83	5.01	7.24	1.05	1.09	1.74	2	1.05	1.09	1.74	2
2vj0	246	8	2.09	2.96	4.12	3.91	3.83	8	21.4	21.43	3.57	5.07	6.5	7.75	2.09	2.96	4.12	3.91
2zjd	121	10	2.35	2.69	2.79	4.6	1.77	1.83	1.91	3.78	1.57	1.58	2.27	3.7	1.57	1.58	1.91	3.7
3bfq	132	15	10.2	11.53	14.22	13.48	1.24	1.41	1.83	2.58	11.81	18.52	21.98	23.03	1.24	1.41	1.83	2.58
3bu3	297	14	6.87	7.06	7.71	8.86	5.77	5.77	10.23	11.07	6.12	10.01	10.01	10.42	5.77	5.77	7.71	8.86
3bwa	276	8	2.42	2.75	3.17	3.94	2.39	2.51	2.98	3.2	1.83	2.7	3.19	3.65	1.83	2.51	2.98	3.2
3cvp	279	6	4.52	4.67	8.47	10.14	3.07	4.97	5.68	7.59	3.08	4.25	5.49	8.02	3.07	4.25	5.49	7.59

receptor pdb id	receptor length (AA)	peptide length (AA)	Run 1 all	Run 1 top 1k	Run 1 top 100	Run 1 top 10	Run 2 all	Run 2 top 1k	Run 2 top 100	Run 2 top 10	Run 3 all	Run 3 top 1k	Run 3 top 100	Run 3 top 10	best from all runs all	best from all runs top 1k	best from all runs top 100	best from all runs top 10
3d1e	366	6	4.39	6.59	8.18	18.82	3.44	3.44	9.96	8.93	2.57	2.57	9.88	12.21	2.57	2.57	8.18	8.93
3d9t	95	6	3.56	4.34	7.25	10.06	3.12	4.28	7.25	9.16	3.02	3.91	11.05	15.91	3.02	3.91	7.25	9.16
**MEAN**	**204.81**	**9.17**	**4.41**	**5.53**	**7.32**	**9.11**	**4.38**	**5.32**	**7.13**	**8.52**	**4.38**	**5.71**	**7.49**	**9.26**	**3.61**	**4.32**	**5.62**	**6.90**

Table S2. CABS-dock performance for 68 unbound cases (listed in rows) in 3 independent prediction runs (shown in separate columns). The table shows the lowest ligand-RMSD values (calculated on the peptide only after superimposition of the receptor structures) among: all 10,000 models (all), top 1000 models (top 1k, selected during filtering and clustering procedure), top 100 models (top 100, selected through further clustering) and top 10 final models (top 10). Last column shows the lowest RMSD values obtained in three prediction runs.

pdb	receptor	peptide	run 1				run 2				run 3				best from 3 runs			
code	length (AA)	length (AA)	all	top 1k	top 100	top 10	all	top 1k	top 100	top 10	all	top 1k	top 100	top 10	all	top 1k	top 100	top 10
1alv	173	11	1.99	2.55	3.6	3.32	2.83	3.24	3.73	4.87	1.68	2.12	3.2	2.47	1.68	2.12	3.2	2.47
1b9k	246	8	3.38	6.89	9.74	9.95	4.07	10.06	14.05	22.2	4.74	6.18	10.07	10.59	3.38	6.18	9.74	9.95
1czz	168	7	2.56	2.56	3.62	3.81	2.03	2.33	2.58	3.21	2.06	2.83	3.15	5.68	2.03	2.33	2.58	3.21
1d1z	104	11	5.26	5.26	7.09	7.48	5.11	5.59	6.85	10.01	5.17	5.33	7.21	8.02	5.11	5.26	6.85	7.48
1eg3	260	13	6.17	10.8	14.45	16.05	6.67	8.44	12.43	22.77	7.47	9.46	14.72	15.02	6.17	8.44	12.43	15.02
1go5	59	9	3.38	3.92	4.92	5.22	2.89	3.4	3.65	4.66	4.17	4.67	11.15	12.66	2.89	3.4	3.65	4.66
1gy7	122	5	3.73	7.64	13.05	13.73	6.01	7.96	12.23	15.15	4.46	4.59	12.19	13.71	3.73	4.59	12.19	13.71
1h1r	256	12	4.37	4.92	7.09	6.55	4.42	5.04	10.24	10.18	3.86	6.12	7.99	8.03	3.86	4.92	7.09	6.55
1i2h	104	6	2.71	3.33	3.95	4.31	3.77	3.77	8.36	10.09	3.29	3.33	4.64	4.63	2.71	3.33	3.95	4.31
1i7g	267	12	1.68	2.01	2.87	7.9	2.26	2.47	2.78	3.38	1.75	1.83	1.83	1.87	1.68	1.83	1.83	1.87
1ie9	240	12	6.93	11.86	13.99	23.44	4.85	7.75	10.22	12.85	6.88	10.98	12.36	15.34	4.85	7.75	10.22	12.85
1jbe	128	13	4.84	5.44	5.75	6.08	4.52	5.76	5.92	6.44	4.37	4.58	7.97	10.79	4.37	4.58	5.75	6.08
1jwf	140	5	2.17	3.89	4.46	4.7	2.52	2.52	4.94	5.71	2.35	2.46	4.24	5.96	2.17	2.46	4.24	4.7
1jwt	250	6	9.44	14.28	17.25	17.23	8.68	11.8	14.42	15.36	9.8	14.08	17.4	19.47	8.68	11.8	14.42	15.36
1lf7	173	11	5.68	7.47	7.6	7.74	4.1	4.97	4.98	4.83	4.89	5.53	6.87	7.59	4.1	4.97	4.98	4.83
1lvb	229	7	5.73	7.41	13.21	18.56	5.73	13.74	14.39	18.3	7.12	7.12	15.6	17.56	5.73	7.12	13.21	17.56
1m7d	435	8	5.61	6.35	7.4	13.37	5.29	5.84	6.78	13.62	3.94	4.67	5.5	5.89	3.94	4.67	5.5	5.89
1n7e	86	8	4.79	4.79	8.03	11.97	4.08	8.01	11.44	11.26	3.79	6.19	10.49	16.36	3.79	4.79	8.03	11.26
1n83	244	10	1.12	1.13	1.13	2.39	2.73	2.73	3.74	3.84	1.48	3.56	8.3	8.56	1.12	1.13	1.13	2.39
1oew	330	8	7.92	8.65	9.87	10.63	5.72	8.05	8.09	9.18	6.58	6.58	7.84	9	5.72	6.58	7.84	9
1oot	60	11	3.43	4.62	5.97	6.97	4.27	4.97	6.1	6.9	3.97	4.62	5.73	7.12	3.43	4.62	5.73	6.9
1ou9	106	8	3.58	4.21	4.97	6.78	3.64	3.64	4.93	11.23	3.25	4.56	4.81	5.18	3.25	3.64	4.81	5.18
1pyw	369	15	3.62	3.65	3.79	4.68	4.62	5.23	5.54	5.8	3.93	5.5	5.55	6.33	3.62	3.65	3.79	4.68
1qbh	105	8	4.72	7.01	9.44	13.51	5.44	6	8.96	12.8	6.09	6.09	8.51	10.44	4.72	6	8.51	10.44
1r6j	164	5	2.52	2.52	8.26	9.3	3.21	3.23	5.43	8.31	3.2	3.72	4.94	8.47	2.52	2.52	4.94	8.31
1rwz	245	11	6.17	10.34	11.2	23.02	4.71	4.71	8.11	19.96	4.73	4.89	6.67	7.61	4.71	4.71	6.67	7.61
1tq3	115	6	1.4	1.4	3.57	6.44	3.58	5.63	7.34	7.39	2.62	2.62	4.7	9.68	1.4	1.4	3.57	6.44
1um5	431	8	3.05	3.05	3.34	3.93	3.54	3.54	5.67	4.78	4.32	4.32	5.89	7.93	3.05	3.05	3.34	3.93
1utn	223	14	7.74	8.46	8.73	12.33	6.95	7.66	8.69	10.88	7.27	7.91	9.31	10.01	6.95	7.66	8.69	10.01
1v49	121	10	2.69	2.69	2.91	4.02	2.19	2.56	2.73	3.45	2.89	2.89	4.23	5.5	2.19	2.56	2.73	3.45
1x2j	290	9	3.92	5.08	5.42	6	3.44	3.68	5.17	5.89	3.57	4.92	5.81	6.54	3.44	3.68	5.17	5.89

pdb code	receptor length (AA)	peptide length (AA)	run 1				run 2				run 3				best from 3 runs			
			all	top 1k	top 100	top 10	all	top 1k	top 100	top 10	all	top 1k	top 100	top 10	all	top 1k	top 100	top 10
1y0m	55	10	3.92	4.97	5.73	7.03	3.53	4.83	5.45	6.21	4	5.24	5.84	8.13	3.53	4.83	5.45	6.21
1yej	442	7	4.67	5.2	10.97	19.17	5.31	6.37	8.26	12.12	4.05	5.94	8.05	7.67	4.05	5.2	8.05	7.67
1z1m	88	9	2.76	3.46	3.72	4.68	3.31	3.48	5.26	4.54	3.39	3.52	3.75	3.62	2.76	3.46	3.72	3.62
1z9l	238	9	4.24	5.32	6.68	8.65	4.18	4.87	5.84	6.91	3.8	4.84	4.91	5.08	3.8	4.84	4.91	5.08
2aa2	253	12	1.84	2	2.95	3.12	2.32	2.52	2.54	2.95	1.73	2.39	2.85	4.12	1.73	2	2.54	2.95
2abx	74	13	6.14	7.58	10.07	10.19	6.5	7.22	7.96	8.76	8.02	9.53	10.21	11.18	6.14	7.22	7.96	8.76
2alf	164	6	1.74	2.42	2.7	4.1	2.17	2.29	3.38	3.46	1.8	1.81	2.75	2.55	1.74	1.81	2.7	2.55
2am9	250	10	1.52	1.58	1.69	2.14	1.57	1.93	2.6	2.22	1.21	1.76	2.36	2.66	1.21	1.58	1.69	2.14
2b9f	337	12	2.44	2.66	3.52	3.86	3.85	3.85	6.1	12.32	2.33	3.62	4.09	4.16	2.33	2.66	3.52	3.86
2bz6	239	15	8.03	16.66	18.28	22.88	7.76	16.35	21.5	23.07	6	9.88	10.72	10.51	6	9.88	10.72	10.51
2ds7	41	6	5.64	5.64	11.31	11.24	5.44	7.26	10.81	11.85	3.63	7.09	10.38	12.17	3.63	5.64	10.38	11.24
2dvj	57	9	2.82	3.29	5.09	7.7	2.96	3.3	5.85	6.15	3.35	5.1	6.38	7.2	2.82	3.29	5.09	6.15
2e45	33	10	4.43	5.35	7.11	17.03	3.38	5.57	7.04	14.19	4.07	7.35	7.97	10.34	3.38	5.35	7.04	10.34
2f1w	137	7	2.82	7.32	7.68	7.82	3.36	4.93	5.65	6.91	3.32	3.35	3.35	7.82	2.82	3.35	3.35	6.91
2fgq	332	8	7.95	14.74	15.97	17.58	10.19	13.38	16.67	17.32	7.73	13.64	14.87	14.82	7.73	13.38	14.87	14.82
2g6f	64	8	3.44	3.44	6.45	6.64	3.25	4.5	5.08	4.94	3.26	5.11	6.14	7.71	3.25	3.44	5.08	4.94
2h14	304	5	2.55	2.94	3.4	4.26	2.57	3.09	3.09	4.7	2.28	2.28	3.73	4.42	2.28	2.28	3.09	4.26
2h3l	95	9	4.36	4.66	6.77	12.2	4.96	4.96	14.76	14.9	3.54	4.71	4.71	9.11	3.54	4.66	4.71	9.11
2hpj	100	5	1.64	3.33	3.83	6.03	2.1	2.17	3.37	4.58	2.35	3.18	3.77	4.91	1.64	2.17	3.37	4.58
2hwq	258	10	2.25	2.82	3.31	2.78	1.16	1.6	2.29	2.68	1.17	1.17	1.36	1.49	1.16	1.17	1.36	1.49
2i3i	95	6	3.85	6.18	7.06	15.82	4.03	4.03	6.82	17.23	3.36	8.39	9.27	9.4	3.36	4.03	6.82	9.4
2iog	238	9	9.98	10.51	14.36	23.02	8.77	10.68	13.32	21.29	9.46	10.35	15.22	20.05	8.77	10.35	13.32	20.05
2j2i	266	8	3.06	4.03	10.45	11.4	3.75	6.46	8.73	11.68	3.92	6.3	9.38	11.1	3.06	4.03	8.73	11.1
2j6k	57	8	3.27	3.8	4.07	7.3	2.98	3.39	3.39	5.62	2.77	3.06	3.34	4.03	2.77	3.06	3.34	4.03
2o9s	67	10	3.24	3.86	5.01	6.96	3.23	4.87	5.68	6.36	3.92	4.36	5.48	6.85	3.23	3.86	5.01	6.36
2qbh	95	6	5.11	5.99	9.29	10.33	5.21	6.29	14.34	14.67	5.03	5.03	7.98	12.62	5.03	5.03	7.98	10.33
2qhn	264	5	4.9	6.27	7.28	9.16	3.74	3.87	7.1	5.22	4.62	5.12	8.55	9.79	3.74	3.87	7.1	5.22
2rtm	120	6	4.44	5.13	6.41	6.93	5.28	5.57	5.96	6.79	5.09	5.25	6.2	6.49	4.44	5.13	5.96	6.49
2yql	60	10	4.57	4.99	6.49	7.59	3.29	3.29	7.69	9.57	6.5	7.18	8.39	13.12	3.29	3.29	6.49	7.59
3d1g	366	6	2.73	2.73	7.04	9	7.61	14.02	14.54	15.3	3.79	6.23	11.18	6.88	2.73	2.73	7.04	6.88
3ekk	297	14	8.03	9.61	10.36	11.6	6.91	7.01	7.11	8.56	4.41	6.57	10.22	9.96	4.41	6.57	7.11	8.56
3hau	198	7	8.66	10.03	10.04	11.73	6.24	6.24	6.8	7	8.11	8.34	8.36	9.08	6.24	6.24	6.8	7
3nsq	211	10	3.19	4.38	6.44	10.29	2.82	3.91	4.37	8.73	2.4	2.64	3.76	5.76	2.4	2.64	3.76	5.76
3pom	337	10	3.59	3.59	3.59	6.69	3.99	4.61	5.26	8.79	2.17	2.17	3.89	6.42	2.17	2.17	3.59	6.42
3rdh	224	14	3.79	4.13	6.16	6.95	3.58	3.93	4.24	6.28	3.88	3.91	4.88	4.96	3.58	3.91	4.24	4.96

pdb code	receptor length (AA)	peptide length (AA)	run 1				run 2				run 3				best from 3 runs			
			all	top 1k	top 100	top 10	all	top 1k	top 100	top 10	all	top 1k	top 100	top 10	all	top 1k	top 100	top 10
3siq	97	7	5.08	5.08	9.61	10.19	7.67	7.67	8.73	15.02	8.65	8.65	8.65	14.31	5.08	5.08	8.65	10.19
3tx7	240	14	4.01	5.77	11.11	12.75	4.14	5.07	5.28	8.25	3.22	3.69	8.73	12.59	3.22	3.69	5.28	8.25
**MEAN**	**191.71**	**9.07**	**4.25**	**5.52**	**7.33**	**9.53**	**4.37**	**5.58**	**7.40**	**9.59**	**4.24**	**5.34**	**7.21**	**8.63**	**3.68**	**4.49**	**6.14**	**7.32**

Table S3. PDB codes of receptor pairs (in bound and unbound form).

bound form pdb code	unbound form pdb code
1awr	2alf
1ce1	1um5
1cka	2dvj
1czy	1czz
1d4t	1d1z
1ddv	1i2h
1eg4	1eg3
1er8	1oew
1gyb	1gy7
1hc9	2abx
1jbu	2bz6
1jd5	1qbh
1jwg	1jwf
1kl3	2rtm
1klu	1pyw
1lvm	1lvb
1mfg	2h3l
1n7f	1n7e
1nq7	1n83
1nvr	2qhn
1nx1	1alv
1oai	1go5
1ou8	1ou9
1pz5	1m7d
1rxz	1rwz
1se0	3siq
1sfi	1utn
1ssh	1oot
1t4f	1z1m
1t7r	2am9
1tp5	1tq3
1tw6	2i3i
1vzq	1jwt
1w9e	1r6j
1x2r	1x2j
1yuc	3tx7
1ywo	1y0m
1z9o	1z9l
2a3i	2aa2
2ak5	2g6f
2b1z	2iog
2b9h	2b9f
2c3i	2j2i
2cch	1h1r
2ds8	2ds7
2fgr	2fgq
2fmf	1jbe
2fnt	3hau
2foj	2f1w
2fvj	2hwq
2h9m	2h14
2ho2	2e45
2hpl	2hpj
2ipu	1yej
2j6f	2j6k
2o02	3rdh
2o4j	1ie9
2o9v	2o9s
2p1t	3nsq
2p54	1i7g
2puy	2yql
2qos	1lf7
2r7g	3pom
2vj0	1b9k
2zjd	1v49
3bu3	3ekk
3d1e	3d1g
3d9t	2qbh
